# Diallyl Trisulfide and Cardiovascular Health: Evidence and Potential Molecular Mechanisms

**DOI:** 10.3390/ijms25189831

**Published:** 2024-09-11

**Authors:** Jovana Novakovic, Maja Muric, Jovana Bradic, Galina Ramenskaya, Vladimir Jakovljevic, Nevena Jeremic

**Affiliations:** 1 Department of Pharmacy, Faculty of Medical Sciences, University of Kragujevac, 34000 Kragujevac, Serbia; jovanabradickg@gmail.com (J.B.); nbarudzic@hotmail.com (N.J.); 2 Center of Excellence for the Study of Redox Balance in Cardiovascular and Metabolic Disorders, Faculty of Medical Sciences, University of Kragujevac, 34000 Kragujevac, Serbia; majanikolickg90@gmail.com (M.M.); drvladakgbg@yahoo.com (V.J.); 3 Department of Physiology, Faculty of Medical Sciences, University of Kragujevac, 34000 Kragujevac, Serbia; 4 I.M. Sechenov First Moscow State Medical University (Sechenov University), 119991 Moscow, Russia; ramenskaya_g_v@staff.sechenov.ru; 5 Department of Human Pathology, I.M. Sechenov First Moscow State Medical University (Sechenov University), 119991 Moscow, Russia

**Keywords:** allitridin, garlic, heart diseases, hydrogen sulfide, polysulfide

## Abstract

Traditionally, garlic has a valuable role in preventing and reducing the incidence of many diseases and pathophysiological disorders. Consequently, some researchers have focused on the beneficial cardiovascular properties of diallyl trisulfide (DATS), the most potent polysulfide isolated from garlic. Therefore, in this review, we collected the available data on DATS, its biochemical synthesis, metabolism and pharmacokinetics, and gathered the current knowledge and the role of DATS in cardiovascular diseases. Overall, this review summarizes the cardioprotective effects of DATS and brings together all previous findings on its protective molecular mechanisms, which are mainly based on the potent anti-apoptotic, anti-inflammatory, and antioxidant potential of this polysulfide. Our review is an important cornerstone for further basic and clinical research on DATS as a new therapeutic agent for the treatment of numerous heart diseases.

## 1. Introduction

Cardiovascular diseases (CVDs) are the leading cause of premature death and severe chronic disability worldwide, accounting for approximately 45% of all deaths in Europe [1]. Although the recent expansion of clinical investigations has produced several effective drugs for the treatment of CVDs, the long-term prognosis and control of complications remain poor. Considering the growing number of clinical patients and the inadequacies of current medical treatment, there is a strong incentive to find more effective targets to treat, mitigate, or prevent this heterogeneous group of diseases [2].

Whole foods and their derivatives were used in the treatment of numerous diseases long before the era of conventional medicine. The traditional medical use of the genus Allium (family Alliaceae/Liliaceae), especially garlic, dates back to ancient times, and in addition to the fresh plant, other forms, such as dried powders, oils, extracts, infusions, and plasters, are currently growing in popularity [3]. Numerous clinical and experimental studies have established a correlation between garlic consumption and a reduced incidence of various diseases and pathophysiological disorders, supporting its antitumor [4,5], antioxidant [5,6], antimicrobial [7,8], anti-inflammatory [9,10], and neuroprotective effects [11]. Various review studies summarizing the effects of functional components in the genus *Allium* indicate that garlic and its supplements can significantly improve cardiovascular risk factors, including blood pressure, cholesterol levels, and inflammatory markers, suggesting their potential role in the prevention and treatment of CVDs [12,13,14].

The beneficial effects of garlic on the cardiovascular system are primarily attributed to its sulfur compounds, which enhance the bioavailability of hydrogen sulfide (H_2_S) [15,16]. Since the valuable effects of dietary garlic on CVD prevention and regression may be partially mediated by H_2_S, it is hypothesized that diallyl trisulfide (DATS), the most potent polysulfide isolated from garlic, plays a key role in improving cardiovascular health [16].

This review summarizes the current state of knowledge and the role of DATS in heart diseases, focusing on recent findings from various animal studies, a better understanding of the potential therapeutic applications of DATS, and molecular mechanisms involved in its cardioprotection.

## 2. DATS and Its Biochemical Synthesis

As previously mentioned, *Allium* species contain a large number of sulfur compounds as well as steroidal saponins, flavonoids, various enzymes, amino acids, and minerals. The sulfur compounds are responsible for the pungent odor of these plants and many of their medical effects. Keeping in mind that garlic contains a higher concentration of sulfur (more than 33 compounds) than any other *Allium* species [17], it is clear why it is considered as one of the most effective disease-preventive foods [18].

**Figure 1:**
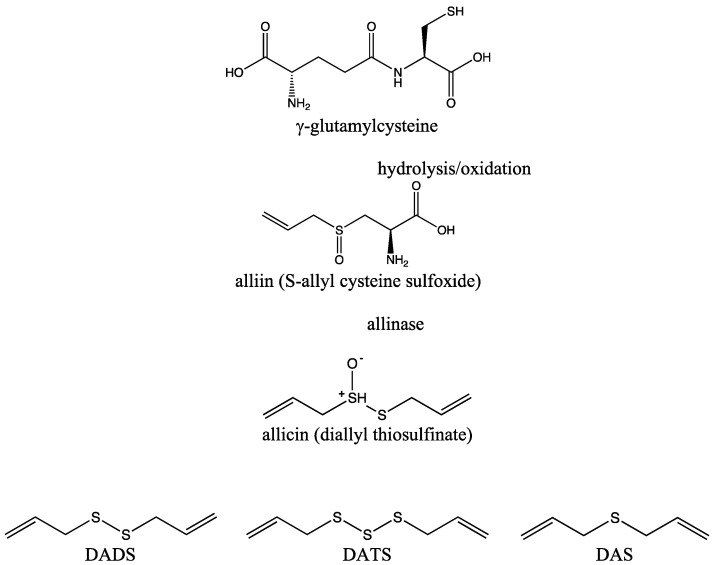
The synthesis pathway of organosulfur compounds (H_2_S donors) isolated from garlic.

Raw intact garlic bulbs contain high amounts of γ-glutamylcysteine, which can be hydrolyzed or oxidized to form an inactive cysteine derivative called alliin (S-allyl cysteine sulfoxide). Garlic bulb injury caused by cutting, crushing, or ingesting activates allinase (alliin lyase), an enzyme that converts alliin to allicin (diallyl thiosulfinate) via the formation of allyl sulfenic acid [19]. The formation of allicin is extremely fast, with almost half of the alliin converted to allicin within 6 s, followed by the release of pyruvic acid and ammonia [20]. Furthermore, allicin is a highly active and unstable odorless metabolite that is susceptible to further oxidation and immediately decomposes into sulfur-containing compounds in addition to allyl sulfides, such as S-allyl mercapto cysteine, vinyl dithiins, and ajoene [16]. The allyl sulfides isolated from garlic differ from each other only in the number of sulfur atoms, which split into two-terminal allyl groups, the most commonly occurring being diallyl disulfide (DADS), DATS, and diallyl sulfide (DAS) [21] (Figure 1). The proportions of these organosulfur compounds in garlic vary depending on the source, extraction methods, age, and several other factors [22,23], with approximately 1000 μg/g DATS found in a garlic bulb [24].

The reactivity of the sulfur compounds is directly related to the number of sulfur atoms present in the molecule. With three sulfur atoms in its structure, DATS is considered the strongest organosulfur compound isolated from garlic. It is more stable than other widely used chemical sources of H_2_S (NaHS and Na_2_S) and it releases H_2_S slowly over time [25,26].

DATS is also known as allitridin, or 4,5,6-trithia-1,8-nonadiene, or under the systematic name di-(2-propenyl) trisulfide with S (SCH_2_CH=CH_2_)_2_ formula [27]. In the present literature, DATS is also listed under other names, such as allitridi, allitridum, allyl trisulfide, and dasuansu [28]. Due to its chemical structure, it is classified as an aliphatic alkene trisulfide [27]. This yellow oil is volatile, has a distinctly unpleasant odor, is soluble in acetone, and is slightly soluble in ethanol (3 mg/mL), dimethyl sulfoxide (5 mg/mL), and 2-propanol and dimethylformamide (10 mg/mL) [28,29].

## 3. Metabolism, Pharmacokinetics, and Potential Side Effects of DATS

To date, almost 480 scientific papers (both reviews and original articles) on DATS have been published in the PubMed database. However, not all the characteristics of DATS have been defined, so further research is needed to determine all the properties of this polysulfide.

Allyl mercaptan, allyl methyl sulfide (AMS), allyl methyl disulfide (AMDS), DAS, and DADS were detected in human breath immediately after consumption of raw garlic and commercial garlic products. In addition, metabolites, such as N-acetyl-S-(2-carboxypropyl)-cysteine, N-acetyl-S-allyl cysteine, and hexahydrohippuric acid have been detected in human urine by high-performance liquid chromatography (HPLC), gas chromatography (GC), mass spectrometry (MS), and their combinations [30,31,32]. Great efforts have been made to measure the levels of some allyl sulfides and other garlic metabolites in vivo. These methods have low sensitivity and selectivity required for the direct determination of DATS in vivo, and they are applied to all sulfur compounds and are not specific to DATS solely. Despite the numerous publications on detection methods, the metabolism of DATS remains unclear. As a highly unstable compound, DATS is rapidly metabolized in the liver via first-pass metabolism, just like many other organosulfur compounds [33]. After ingestion, DATS is converted to other sulfur-containing metabolites, limiting the unmetabolized amount entering the systemic circulation [33,34].

There are only a few studies that have investigated the pharmacokinetics of DATS, but it is well-known that hydrophobicity, a brief half-life, lack of target selectivity, and low bioavailability may limit DATS effectiveness [35]. *Sun* and coworkers determined the DATS concentration in rat blood using GC with electron-capture detection and identified its major metabolite with GC-MS. They concluded that it is difficult to determine the levels of DATS due to its volatility, ultraviolet (UV) end-absorption, and instability. The DATS content was significantly reduced when the organic solvent was evaporated to dryness under a nitrogen stream. The direct addition of acetonitrile to blood samples prevented the degradation and evaporation of DATS during centrifugation and plasma separation [28].

After two weeks of DATS treatment (10 mmol/kg body weight/day intraperitoneally), a significant increase in quinone reductase, glutathione peroxidase, and glutathione S-transferase activity in rats was observed [36]. Similarly, DATS (89 mg/kg/day) administered orally to rats for six consecutive days significantly increased quinone reductase and glutathione S-transferase activity in a variety of tissues [37]. Glutathione S-transferase activity was also significantly increased after 6 weeks of DATS consumption (70 mg/kg three times a week) in rats, while N-nitrosodimethylamine demethylase activity was decreased [38]. Literature data indicate that GSH-rich human red blood cells can initiate the release of H_2_S from DATS. Biological thiols, especially exofacial membrane protein thiols with trans-plasma membrane reductase systems, play an important role in the production of H_2_S. NADH, NADPH, and GSH maintain the level of biological thiols, thereby preserving H_2_S production [39]. Since blocking the thiol group in membrane proteins does not completely prevent the production of H_2_S, DATS passes through the membrane and interacts with intracellular GSH, resulting in the synthesis of H_2_S [40,41,42].

The addition of DATS at a concentration of 50–500 μM to a GSH solution leads to the immediate production of H_2_S, while the addition of DADS at the concentration of 50 μM to the GSH solution does not trigger any detectable production of H_2_S. According to Liang and coworkers, DADS likely reacts with GSH by thiol-disulfide exchange to generate allyl mercaptan and S-allyl glutathione disulfide, whereas there are two possible nucleophilic thiol-disulfide attacks on GSH for DATS. Allylic sulfur generates S-allyl glutathione disulfide and allyl perthiol. Reduction with GSH or the central sulfur atom of DATS leads to the formation of allyl mercaptan and S-allyl glutathione trisulfide with the possible release of H_2_S. Subsequently, these compounds can be reduced in the presence of GSH and release H_2_S [43].

In summary, DATS interacts with thiol groups or thiol-containing compounds from biological systems and increases the bioavailability of H_2_S, which plays a crucial role in providing beneficial effects on overall health [16,19,20,22]. As the various cardioprotective effects of H_2_S have been extensively studied, the role of DATS, one of the most potent naturally occurring H_2_S donors, in cardiovascular health is of increasing interest.

Although garlic is ”generally recognized as safe”—GRAS by the U.S. Food and Drug Administration, certain precautions should be followed to reduce the risk of side effects [44]. Current evidence suggests that chronic administration of garlic oil enriched with DATS prolongs bleeding time and thrombin time, along with increases in antithrombin III and protein C activity. These effects suggest that DATS has anticoagulant properties, which could increase bleeding risk or risk from hemorrhage, especially if consumed in higher doses in individuals with underlying coagulopathies or those prescribed with anticoagulant medications [45]. For that reason, many surgeons recommend their patients to avoid consumption of large amounts of garlic 7 to 10 days before surgery, as prolonged bleeding has been recently reported in a patient with spontaneous epidural hematoma [14].

Considering the abovementioned facts, the concentration of DATS in treatments should be carefully monitored to avoid potential adverse effects on the hemostatic balance associated with garlic [46]. In healthy volunteers, garlic ingestion may cause variable effects on the pharmacokinetics of anticoagulants, while in combination with protease inhibitors, it can cause gastrointestinal toxicity [47]. In his recent paper, Persaud described a female patient with iron-deficiency anemia, probably caused by excessive ingestion of garlic [48]. Additionally, garlic may cause undesirable effects such as burning sensations, diarrhea, gastrointestinal discomfort, and mucosal irritation [14,49], while its allergic manifestations, including asthma and contact dermatitis, have also been reported among individuals exposed to garlic dust and powders [50,51]. In an experimental study, researchers demonstrated that a low dose of garlic (50 mg/kg/day) had no significant effect on liver and lung tissues. However, when garlic is administered in higher doses (250, 500, and 1000 mg/kg/day), dose-dependent toxicity occurs in these organs, while the highest dose of garlic (1000 mg/kg/day) also induced focal injuries of hepatocytes [52]. Recent experimental findings suggest that, while administering relatively high doses of garlic aqueous extract can provide beneficial effects in rats, it also leads to significant tissue damage, particularly in the kidneys and lungs [53]. These observations highlight the necessity of restricting the dosage of medical herbs, such as garlic, to a safe and controlled range to mitigate potential adverse effects while maximizing their therapeutic benefits [53].

The current literature predominantly highlights the potential adverse effects of garlic, while knowledge regarding DATS, one of its most powerful compounds, remains limited. On the other hand, while the cardioprotective features of DATS should not be neglected, we encourage additional preclinical and clinical studies to comprehensively elucidate its safety. It would be of particular importance to determine the exact safe and effective dosage for DATS use in humans in order to achieve the cardioprotective effects of this compound. Moreover, it is crucial to explore potential interactions between DATS and various foods, vitamins, drugs, and other substances.

## 4. Effects of DATS on Cardiac Structure and Function in Experimental Models

Since Abe and Kimura reported the potential physiological role of H_2_S, interest in the physiological signaling effects of this simplest intracellular thiol has increased [54,55]. Given the pleiotropic protective effects of H_2_S in the cardiovascular system, an increasing number of animal studies have been conducted in recent years to investigate the cardioprotective effects of agents capable of releasing H_2_S [56,57]. DATS has been shown to improve cardiovascular structure and function in various animal models, including models of myocardial infarction (MI), diabetic cardiomyopathy, and hypertensive cardiomyopathy (Table 1).

## 5. DATS in Diabetic Cardiomyopathy

Diabetes mellitus (DM) is the leading risk factor for CVDs, and diabetic cardiomyopathy stands as one of the most common cardiovascular complications of diabetes. It initially manifests as myocardial fibrosis, dysfunctional rearrangement, and symptomatic diastolic dysfunction, and later as systolic dysfunction and cardiac hypertrophy leading to symptomatic heart failure. Numerous abnormalities in myocardial tissue, including impaired insulin metabolic signaling, mitochondrial and microvascular dysfunction, increases in oxidative stress (OS), inflammation and glycation, and a decrease in nitric oxide (NO) bioavailability, have been identified, while the molecular mechanisms underlying observed pathophysiological changes are complex and poorly understood [70,71].

Garlic oil and DATS in particular have been shown to increase insulin secretion and sensitivity and therefore provide effective glycemic control in streptozotocin (STZ)-induced diabetic rats [72]. In 2013, Huang and coworkers investigated the effects of DATS on diabetic cardiomyopathy in rats. After 16 days of treatment, DATS significantly improved cardiac function and restored contractile function to physiological levels. The authors further suggested that DATS consumption alleviated diabetic cardiomyopathy by reducing OS and apoptosis, along with promoting survival pathways [58]. Furthermore, DATS administration also had cardioprotective effects in STZ-induced diabetic rats with ischemia/reperfusion (I/R) injury [73].

Since long-standing diabetes and accompanying diabetic cardiomyopathy result in heart failure development, the beneficial effects of DATS on the prevention of cardiovascular complications are certainly of great importance. Previously published work by our group has shown that DATS consumption significantly improves recovery of cardiac function and attenuates cardiac remodeling after ex vivo-induced I/R injury in diabetic rats. Our findings indicated that DATS significantly improved the contractility index and provided sufficient contractile reserve to ameliorate the adverse effects of diabetes on cardiac contractility [63]. These results were also confirmed in another study in which diabetic rats with left coronary artery (LCA) ligation-induced MI were examined, and the authors reported an association between DATS consumption and an increase in ejection fraction (EF) [59].

## 6. DATS in Hypertension and Hypertrophic Cardiomyopathy

Along with diabetes, hypertension is one of the main causes of various CVDs, including coronary heart disease and atrial fibrillation. Prolonged pressure overload leads to the development of left ventricular (LV) hypertrophy, which, together with cardiac fibrosis, results in progressive dysfunction of the cardiac muscle and consequently to the development of heart failure [74]. An experimental study showed that intraperitoneal administration of DATS (7.5 or 15 mg/kg) over 8 weeks had the potential to reduce the remodeling of hypertrophic ventricular myocytes in spontaneously hypertensive rats (SHR) [60]. To our knowledge, this is the only study that has demonstrated the cardioprotective effects of DATS in an animal model of hypertension. On the other hand, our group performed an experimental study to investigate the effects of DATS in the treatment of metabolic syndrome and its associated manifestations. Since one of the features of metabolic syndrome is hypertension, we found that DATS significantly lowered diastolic blood pressure and heart rate [64]. These beneficial effects are thought to be caused by H_2_S production and a reduction in peripheral vascular resistance [75,76]. As there is insufficient evidence of a beneficial effect on hypertension and hypertrophic cardiomyopathy, it is important to conduct further research in this field to confirm the protective effect in various diseases associated with hypertension.

## 7. DATS in Myocardial Infarction

Myocardial I/R triggers irreversible cardiac damage that occurs both during vascular occlusion and during restoration of blood flow recovery. The factors contributing to I/R injury are very complex and include microvascular dysfunction, the release of reactive oxygen species (ROS), disruption of K^+^ and Ca^2+^ concentrations, a complex inflammatory response, and activation of mitochondrial apoptosis and necrosis [77,78,79].

Damage to the heart during I/R injury can be prevented by ischemic preconditioning, which involves short I/R episodes before a long I/R phase. The common goal of most preclinical studies addressing I/R injury is to identify and develop agents that can prevent cardiac damage more effectively than preconditioning alone [80]. Several experimental studies have reported the preconditioning potential of DATS on the heart [61,62,63,69].

The first study to present DATS as a potential preconditioning agent was the investigation by Zhang and coworkers, who hypothesized that DATS mimics the effects of ischemic preconditioning via a protein kinase C (PKC)-mediated mechanism. The authors observed that DATS can precondition the heart and reduce infarct size by reducing left ventricular systolic pressure (LVSP) during DATS infusion. They also showed that DATS can mimic the effects of ischemic preconditioning probably by binding to receptors such as adenosine, NO, or others, thereby activating PKC, which blocks Ca^2+^ influx and triggers heart protection [61].

In a study conducted by Yu and coworkers, DATS was first proposed as a novel therapeutic strategy against I/R injury in the diabetic state. The authors showed that DATS induced cardioprotection in diabetic rats by limiting infarct size and improving cardiac contractile function, mainly by attenuating cellular apoptosis [62].

Despite the major benefits of heart preconditioning, it is difficult to translate this strategy into humans because it must be initiated before I/R injury, and MI is rarely predictable. Therefore, a more feasible alternative, ischemic postconditioning, which includes several short I/R cycles after prolonged ischemia, was quickly introduced [81]. In recent years, DATS has been increasingly shown to have a cardioprotective effect as a postconditioning agent [65,66,67,69].

In 2012, Predmore and coworkers demonstrated that 16 days of DATS consumption could reduce myocardial infarct size and improve both fractional shortening (FS) and EF in rats with acute I/R injury induced by LCA ligation [65]. In addition, the effects of DATS were also studied in an experimental model of heart failure induced by transverse aortic constriction (TAC). DATS therapy resulted in less LV dilation and dysfunction and reduced the progression of perivascular and intermuscular fibrosis and cardiac hypertrophy [67]. To improve bioavailability and thus efficiency, Wang and coworkers [66] synthesized mesoporous iron oxide nanoparticles (MIONs) with DATS, which showed low organ toxicity, excellent biodegradability, and great potential to attenuate I/R-induced heart injury in mice.

Bearing in mind that ventricular arrhythmias are the leading cause of death in patients with myocardial ischemia and MI [82], the cardioprotective effects of DATS were also investigated using monophasic action potential (MAP) technology, which recorded the line graphics of depolarization to repolarization of transmembrane single-cell action potentials in vitro in rats with MI caused by LAD ligation. DATS was shown to be effective in slowing His bundle (A-H, H-V) conduction and sinus rhythm. DATS administration was also linked with a slowing of atrioventricular and sinus node conduction velocities and an increased rate of the effective refractory period (ERP) and monophasic action potential duration 90 (MAPD_90_) in hearts of healthy rats with induced MI. The observed antiarrhythmic potential of DATS is probably caused by blocking the ion channels, which will be discussed in detail in the next section [61].

In addition to the aforementioned myocardial injury models, the role of DATS was also investigated in an experimental model of heart transplantation, as acute myocardial injury and congestive heart failure are very common in heart transplant recipients. Heart transplantation is the gold standard life-saving surgical procedure in which a damaged or diseased heart is replaced with a healthy donor heart. One of the main challenges for successful heart transplantation is reducing the extent of I/R damage by shortening the preservation time and/or improving the quality of the preservation solution [83]. In an experimental heart transplantation study, Sun and coworkers [68] investigated the cardioprotective effects of mesoporous silica nanoparticles (MSN)-DATS in the cardiac preservation solution. They found that DATS-MSN can preserve the heart and improve the survival and long-term function of the allograft.

The above-mentioned studies have shown the many ways in which DATS can be used for cardioprotection through various signaling modifications, which will be discussed in the next sections.

## 8. DATS Dosing for Cardioprotection

Although various experimental studies have proven that DATS can become an important part of the therapeutic strategy for various CVDs, establishing the dose, administration method, and optimal duration of the treatment is still a matter of debate.

As detailed in Table 1 and Table 2, chronic experimental studies evaluating the cardiovascular benefits of DATS in animal models were administered over a duration ranging from 3 to 57 days and predominantly employed dosages ranging from 20 to 40 mg/kg orally, 7.5 to 15 mg/kg intraperitoneally, or 5 mg/kg intravenously. However, the concentration of DATS, treatment duration, and delivery method can vary significantly in different garlic products, including garlic oils, aged garlic extracts, and garlic powders due to variations in preparation methods. Garlic oil, typically extracted through steam distillation, is especially rich in DATS, with concentrations ranging from 1% to 3% of total sulfur compounds [55]. Conversely, aged garlic extract, produced by aging fresh garlic in ethanol, generally contains DATS concentration less than 1% [84]. DATS is also found in garlic powders, which are ground and dried versions of garlic; however, the concentration of DATS in garlic powder can vary significantly depending on storage conditions and drying methods, often leading to lower and less consistent levels of this compound [55]. Since achieving these DATS concentrations in humans would require the ingestion of impractically large amounts of fresh garlic, the use of garlic extract, powder, oil, or DATS supplements presents a more feasible method for obtaining clinically significant effects. It is worth mentioning that studies investigating different DATS formulations to improve its stability, bioavailability and therapeutic efficacy are very limited. Zhang et al. demonstrated that the use of DATS-loaded liposomes not only protected DATS from degradation, but also enhanced its absorption and retention in the body, which could be useful for therapeutic applications [85]. Similarly, another study by Alrumaihi et al. explored a formulation of DATS in nanoparticles, which showed promise in improving its solubility and controlled release [86]. These advances in pharmaceutical formulations could potentially overcome the limitations associated with the dosage of garlic-based preparations, and the low bioavailability and rapid metabolism of DATS.

## 9. Potential Molecular Mechanisms Responsible for Cardioprotective Effects of DATS

Given the numerous preclinical studies that have demonstrated the efficacy of DATS in various animal models, it is crucial to investigate the key mechanisms underlying its cardioprotective potential. As there is increasing evidence that H_2_S plays a protective role in the progression of CVDs, DATS may exert its cardioprotective effects at least in part via H_2_S synthesis. Given the high prevalence of CVDs, there is an urgent need to uncover the possible underlying mechanisms of DATS-mediated cardioprotection. Understanding its mode(s) of action could provide novel insights into the beneficial effects and possible therapeutic implementation of DATS as a major H_2_S donor [65]. Therefore, in the following sections, we attempt to highlight the most discussed molecular mechanisms and signaling pathways associated with the cardioprotective effects of DATS (Table 2).

### 9.1. Antioxidative Effects of DATS

It is well known that OS, as a state of imbalance between pro- and antioxidants, is one of the major causes of tissue damage [91]. ROS are highly involved in the pathophysiology of various CVDs, such as myocardial I/R injury, heart failure, and diabetic cardiomyopathy [92]. DATS is the most effective antioxidant among organosulfur compounds of garlic oil. According to the available literature, the antioxidant properties of DATS have been well documented in various experimental studies, mainly related to I/R injury or hyperglycemia in experimental animals or in cell cultures.

**Figure 2:**
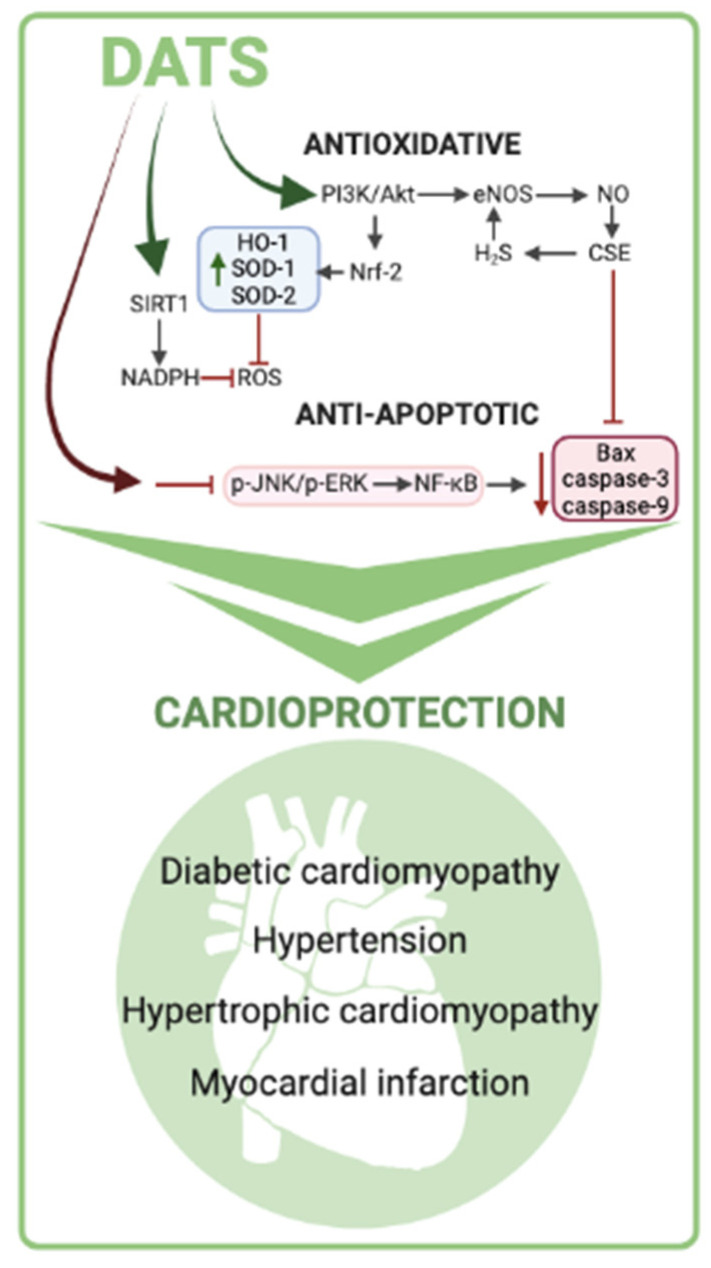
The main antioxidant and anti-apoptotic molecular mechanisms of cardioprotection by DATS.

Several signaling pathways have been proposed as the main drivers of the antioxidant effects of DATS (Figure 2). Nuclear factor erythroid 2-related factor 2 (Nrf2) is a transcriptional factor involved in protective molecular signaling by regulating the expression of various genes involved in the production of antioxidant enzymes. Therefore, targeting Nrf2 could improve the treatment strategy for various diseases caused or exacerbated by the harmful effects of OS. In an experimental study examining cardiomyocytes exposed to hyperglycemia, DATS administration reduced OS primarily by increasing the expression of antioxidants (HO-1, SOD-1, and SOD-2) via upregulation of Nrf2 expression and its translocation to the nucleus. Further investigations showed that DATS-mediated Nrf2 upregulation was driven by another signaling cascade, the phosphatidylinositol 3-kinase/protein kinase B (PI3K/Akt) pathway [88].

The protective role of DATS was also demonstrated in human umbilical vein endothelial cells (HUVEC) incubated with oxidized low-density lipoprotein (LDL), in which this substance improved eNOS activity and production of NO via PI3K/PKB signaling [90]. In addition to H_2_S, another gasotransmitter, NO, plays a key role in cardiovascular health. NO produced by eNOS has been shown to have cardioprotective effects associated with preserved mitochondrial function, attenuation of OS, and increased myocardial vascular density. Furthermore, NO enhances the regulation of cystathionine gamma-lyase (CSE), an enzyme that generates H_2_S, while on the other hand, H_2_S activates eNOS via an Akt-dependent mechanism and increases NO production. Although H_2_S and NO function via independent signaling pathways, the interaction between these two gasotransmitters has become the focus of scientific research in recent years [93]. Cardioprotective effects of DATS were also demonstrated during I/R injury in mice by Predmore and coworkers [65], suggesting that the improvement in myocardial structure and function was mainly driven by H_2_S conservation and promotion of NO bioavailability. In TAC-induced heart failure in mice, administration of DATS increased NO production via activation of the Akt-eNOS-NO signaling pathway and concomitantly increased the expression of antioxidant biomarkers (GPx1 and HO-1) [67].

Another transcription factor involved in various biological processes is nuclear factor kappa B (NF-κB), which can be triggered by numerous pathological stimuli and regulate different signaling pathways. Previously, DATS was reported to inhibit lipopolysaccharide-triggered ROS-mediated activation of NF-κB in mouse lungs and macrophages [94,95]. Treatment with DATS led to a reduction in cell death in hyperglycemia-exposed cardiomyocytes, which was also due to inhibition of the ROS-mediated JNK/NF-κB signaling cascade [59].

Nicotinamide adenine dinucleotide phosphate (NADPH) oxidase is one of the major generators of ROS. The hyperglycemia-induced activity of NADPH oxidase and the resulting excessive mitochondrial ROS generation was reduced by DATS administration, mainly by preserving the antioxidant defense system (SOD and GPx) in obese DM rats [89]. In addition, in our previous study, we investigated the cardioprotective effects of DATS in diabetic rats with induced I/R injury. Chronic administration of DATS improved the redox status (decreased TBARS, NO_2_^−^, O_2_ levels, and increased SOD and CAT levels) in the venous blood of both healthy and diabetic rats [63]. Similar results were obtained in our other study, which involved rats with metabolic syndrome [64].

### 9.2. Anti-Apoptotic Effects of DATS

There is a large body of evidence indicating that DATS has enviable anti-apoptotic effects (Figure 2). In H9c2 cardiomyocytes treated with high amounts of glucose and in the heart tissue of STZ-induced diabetic rats, DATS increased CSE expression, which led to a reduction of pro-apoptotic factors. The researchers hypothesized that the observed effects were mediated by activation of the IGF1R/p-Akt survival pathway [87]. Furthermore, administration of DATS in DM rats with I/R injury reduced apoptosis in cardiac tissue via activation of the AMPK and AKT/GSK-3β/HIF-1 signaling cascade, followed by functional and morphological recovery of the myocardium [62].

Chronic hyperglycemia as a major feature of diabetes mellitus is responsible for the increased production of advanced glycation end products (AGEs), which together with their receptors RAGE and sRAGE are involved in the pathophysiology of heart disease [96]. The protein kinase C delta (PKCδ) signaling cascade has been shown to be associated with reperfusion-induced myocardial injury, in part via its translocation to the mitochondria, leading to impaired energy production, increased mitochondrial ROS formation, and apoptosis [97]. In one study, PKCδ activation and further mitochondrial dysfunction were detected in cardiac cells in response to excessive AGE-mediated ROS production [98]. Furthermore, in diabetic rats treated with DATS for 3 weeks, the relative gene expression of Bcl-2 was increased, while the expression of the pro-apoptotic markers Bax and caspase-3 and -9 was significantly decreased compared to untreated diabetic rats [63]. In a study by Wen and coworkers, DATS treatment reduced doxorubicin-induced DNA damage in cardiomyocytes by mitigating ROS production and apoptosis via activation of the PI3K/Akt signaling pathway [99]. On the other hand, administration of DATS in AGE-induced H9c2 cardiomyoblasts decreased apoptotic proteins, such as cleaved caspase-9, cleaved caspase-3, and cytochrome c. Suspected mechanisms include decreased ROS levels and reduced AGE-dependent mitochondrial PKCδ translocation and PKCδ signaling in cardiomyocytes, including in STZ-induced diabetic rats [98].

Mitogen-activated protein kinase (MAPK) signaling pathways can be activated by various stimuli, such as OS and excessive ROS generation, leading to cell death. In mammals, there are five major MAPKs (ERK 1/2, ERK 3/4, and ERK 5; JNKs 1, 2, and 3; and p 38 isoforms α, β, γ, and δ) [100]. The cardioprotective effect of DATS was demonstrated in a study investigating doxorubicin-induced pro-apoptotic MAPK signaling activation in H92c cardiomyocytes. Administration of DATS decreased ROS-dependent JNK/ERK/NFkB signaling activity, followed by a reduction in pro-apoptotic factors and cardiomyocyte apoptosis [25].

Another signaling pathway has been proposed as a mediator of the cardioprotective DATS effects. Silent information regulator l (SIRT1) appears to be highly involved in the preservation of myocardial damage during I/R injury due to the enhancement of OS and ER stress [62,101]. Moreover, in a study by Yu and coworkers [73], the cardioprotective effect of DATS during I/R injury in diabetic rats was reflected by a reduction in cell death due to decreased OS and endoplasmic reticulum (ER) stress. These results were associated with activation of the SIRT-1 signaling cascade during DATS administration.

### 9.3. Anti-Inflammatory Effects of DATS

In addition to the well-studied antioxidant and anti-apoptotic effects of DATS (Figure 2), some available literature data suggest strong anti-inflammatory properties of this polysulfide isolated from garlic. Some authors state that the anti-inflammatory effect of DATS is expected, since it is known that consumption of *allium* vegetables is associated with anti-inflammatory effects, and that H_2_S can inhibit the Toll-like receptor 4/NF-kB signaling pathway and NLRP3 activation in cardiomyocytes and protect against inflammation [102,103,104]. Also, Lee and coworkers [105], using a RAW 264.7 macrophage model, also suggested DATS suppresses inflammation through the same molecular mechanisms. Using a similar experimental model, You and coworkers [106] suggested that DATS inhibits anti-inflammatory activity in both cell and animal models through the downregulation of AKT1/TGF-β-activated kinase-mediated MAPK and NF-kB signaling pathways.

In agreement with these results, in the previously mentioned studies conducted in our laboratory [63,64], 3 weeks of DATS consumption was shown to significantly alleviate inflammatory markers in rat hearts subjected to I/R injury. However, further experimental evidence is needed to explain the in vivo effects of this polysulfide isolated from *allium* species. However, considering that it is still speculated whether H_2_S has a pro-inflammatory or anti-inflammatory effect [107], more data are needed to further determine the role of the anti-inflammatory effects of DATS in cardioprotection.

### 9.4. Effects of DATS on Mitochondria

Another important question is whether DATS has a protective effect on mitochondria, and if so, which key molecular mechanisms are responsible for this. CVDs are characterized by impaired mitochondrial function, which is reflected in decreased ATP generation and increased ROS production [108]. In addition, mitochondrial fission and fusion have recently attracted much attention from scientists for their involvement in the pathophysiology of CVDs [109]. Dynamin-related protein 1 (Drp1) is an important regulator of mitochondrial fission. According to a recent study, DATS decreased cell death in endothelial cells induced by hyperglycemia, which was mainly caused by decreased mitochondrial fission via decreased expression of Drp1. The proposed mechanism responsible for Drp1-mediated mitochondrial protection involves activation of the AMPK signaling pathway [110]. In previously published studies using an I/R injury model in mice, administration of DATS improved cardiac function and protected the injured myocardium, followed by a reduction in mitochondrial respiration and improved mitochondrial coupling of electrons [65]. Furthermore, in vascular smooth muscle cells (VSMC) exposed to AngII, there was increased phosphorylation of Drp1, which promoted mitochondrial fission and phenotypic changes [111]. However, DATS treatment reversed these vascular changes by decreasing mitochondrial fission due to interference with ROCK1/Drp1 signaling.

Moreover, ROS-mediated disruption of the mitochondrial membrane potential (∆ψ_m_) can be reversed by GSK-3β inhibition, preventing necrosis in cardiac cells [112]. During I/R injury in diabetic myocardium, administration of DATS showed protection of the myocardium by promoting AMPK-mediated activation of AKT/GSK-3β/HIF-1α signaling [62].

The available literature indicates that H_2_S exerts various cytoprotective effects, particularly due to improved mitochondrial function [113]. Therefore, it would be of great importance to perform more sensitive studies that could reveal the relationship between DATS and its H_2_S release and mitochondrial protection.

### 9.5. Effects of DATS on Cardiac Ion Channels

The harmonious activation of various specified cardiac ion channels generates a cellular action potential in the heart, while any disturbance of this physiological ion homeostasis can promote cardiac arrhythmias [114]. According to the available literature, garlic has shown antiarrhythmic effects in various animal studies [20,115]. While DATS is one of the most potent garlic compounds in terms of H2S generation, several researchers have investigated the effects of DATS on the conduction system and ion homeostasis.

In a study investigating the effects of DATS on HEK 293 cells, it was shown that this substance mainly inhibits hKv4.3 channels, as well as hKv1.5 channels, hERG channels, hKCNQ1/hKCNE1 channels with a lower blockade potential, and hKir2.1 channels with the least effect. The inhibition of potassium currents could lead to a prolongation of the action potential duration and consequently to a lower incidence of atrial arrhythmias [59]. Furthermore, administration of DATS in isolated human atrial cardiomyocytes reversibly inhibited the transient outward potassium current (*I*to) and resulted in a prolongation of action potential duration [116].

On the other hand, DATS also decreases *I*to in ventricular myocytes of mice, which has been reported as a dose-dependent effect [116]. The production of an ideal drug for the treatment of ventricular arrhythmias remains a major challenge for pharmaceutical experts. It has been suggested that the ability to prolong cardiac repolarization without a reverse application dependence should be the preferred property of such an arrhythmic drug [117]. The promising results regarding the antiarrhythmic properties of DATS were reported in an experimental model of MI, in which DATS acted on the conduction system without reverse application dependence, similar to amiodarone [69]. DATS has also been shown to directly increase the *I*to current in ventricular myocytes from rats with induced heart failure [118]. Further research in this area could provide crucial insights and improve the treatment strategy in patients with arrhythmias.

## 10. Conclusions and Future Directions

DATS shows promising beneficial effects on cardiovascular health by combining antioxidant, anti-inflammatory, and vasodilatory actions. However, although significant progress has been made in understanding the basic molecular pathways through which DATS exerts its effects, there are several gaps in our knowledge. Therefore, upcoming research should focus further on elucidating the precise molecular targets of DATS. More uniform study designs should be used in future trials, particularly concerning the kind, dosage, and duration of the garlic formula. Given the limited data on DATS in humans, more in-depth clinical studies are needed to determine the optimal treatment duration and dosing strategies for different clinical scenarios, and to explore its efficacy and safety profile in diverse population of patients. Expanding research on DATS may uncover novel therapeutic applications, potentially enhancing its role in cardiovascular disease prevention and treatment. Continued research into its mechanisms and clinical relevance will help define its full potential and safety profile, which will contribute to the development of new interventions aimed at improving the cardiovascular health of people around the world.

## Figures and Tables

**Table 1:** A summary of selected experimental studies reporting DATS effects on cardiac structure and function.

Animal Species	Experimental Model	Route/Dose/Duration	Major Cardiac Effects	Potential Use	Ref.
Male *Wistar albino* rats	Diabetic cardiomyopathy induced by 65 mg/kg of streptozotocin	Per os by gavage, 40 mg/kg body weight, every other day for 16 days	↑ HR ↓ LVESD ↑ LVPWd, LVPWs ↑ FS	Alleviation of diabetic cardiomyopathy	[58]
Male *Wistar albino* rats	Diabetes induced by 65 mg/kg of streptozotocin	Per os by gavage, 40 mg/kg body weight, every other day for 16 days	↑ EF		[59]
Male spontaneously hypertensive rats [SHR]	Isolation of ventricular myocytes	Intraperitoneal injection, 7.5 mg/kg or 15 mg/kg for 8 weeks	↓ LV hypertrophy Recovery of the transient outward potassium current of ventricular myocytes	Alleviation of LV hypertrophy in hypertension	[60]
Male rabbits	Ex vivo induced I/R injury on Langendorff apparatus-30 min ischemia followed by 120 min reperfusion	Acute heart perfusion, 60 μmol/L for 5 min, followed by 10 min drug-free interval before induced I/R injury	↓ LVsp ↓ infarct size	Preconditioning agent	[61]
Male *Sprague Dawley* rats	Diabetes induced by 40 mg/kg of streptozotocin and myocardial infarction by LCA-30 min ischemia followed by 3 h of reperfusion	Per os by gavage, 20 mg/kg body weight 3 days before myocardial I/R injury and once again after 20 min of ischemia	↑ LVsp ↑ +dP/dt max, −dP/dt max ↓ infarct size	Preconditioning agent in diabetic condition	[62]
Male *Wistar albino* rats	Diabetes induced by 60 mg/kg of streptozotocin and ex vivo induced I/R injury on Langendorff apparatus-30 min ischemia followed by 60 min of reperfusion	Per os by gavage, 40 mg/kg body weight, every other day for 21 days	↓ IVSd ↑ FS ↓ LVPWd, LVPWs ↑dp/dt max, dp/dt min ↑ HR ↑ CF ↓ myocardial structure turbulence		[63]
Male *Wistar albino* rats	Metabolic syndrome induced by one month of high fat diet followed with 25 mg/kg of streptozotocin and ex vivo induced I/R injury on Langendorff apparatus-30 min ischemia followed by 60 min of reperfusion	Per os by gavage, 40 mg/kg body weight, every other day for 21 days	↑ FS ↑E F ↓ DBP ↑ dp/dt max, dp/dt min ↓ CF ↓ myocardial structure turbulence	Preconditioning agent in condition of metabolic syndrome	[64]
Male C57 BL6/J mice	Myocardial infarction by LCA ligation-45 min ischemia followed by 24 h of reperfusion	Intravenous injection, 200 μg/kg 5 min before reperfusion; Intraperitoneal injection, 200 μg/kg 22.5 min before reperfusion	↓ infarct size ↓ LVEDD, LVESD ↑ EF ↑ FS	Postconditioning agent	[65]
Male C57 BL6/J mice	Myocardial infarction by LAD ligation-30 min ischemia followed by 24 h of reperfusion	Intravenous injection of MIONs, 0.71 µg/100 g	↑ HR ↑ EF ↑ FS		[66]
Male C57 BL6/J mice	Heart failure by TAC	Intraperitoneal injection, 200 μg/kg 24 h after TAC and for the next 12 weeks/day	↓ LVEDD, LVESD ↑ EF ↓ cardiac hypertrophy ↓ fibrosis		[67]
Male *Sprague Dawley* rats	Heart transplantation model	Preservation solution, 3 µg/mL in MSN 6 h before transplantation	↑ LVDP ↑ dP/dt max ↓ arrhythmia score ↓ time of reanimation ↓% myocardial neutrophilic infiltrate ↓ necrosis ↑ survival rates of heterotopic hearts ↑ EF ↑ FS ↓ LVIDs, LVIDd ↓ fibrotic area	Improving the survival of allografts	[68]
Male *Sprague Dawley* rats	LAD	Acute heart perfusion, 7.5 mg/L For 10 min, followed by 15 min drug-free interval	prolonged ERP and MAPD_90_ ↑ ERP/MAPD_90_ ratio ↓ His bundle (A-H, H-V) conduction ↓ incidences of arrhythmia	Postconditioning and antiarrhythmic agent	[69]

Abbreviations: LCA—left anterior descending coronary artery; LVEDD—left ventricular end-diastolic diameter; LVESD—left ventricular end-systolic diameter; EF—ejection fraction; FS—fractional shortening; MIONs—mesoporous iron oxide nanoparticles; LAD—left anterior descending coronary artery; CF—coronary flow; HR—heart rate; DBP—diastolic blood pressure; TAC—transverse aortic constriction; MSN—mesoporous silica nanoparticles; LVDP—left ventricle developed pressure; ERP—effective refractory period; MAPD_90_—monophasic action potential duration 90; LVsp—left ventricle systolic pressure; IVSd—interventricular septum thickness at end-diastole; LVPWd—left ventricular posterior wall thickness at end-diastole; LVPWs—left ventricular posterior wall thickness at end-systole; ↑—increased; ↓—decreased.

**Table 2:** Summary of the key molecular mechanisms driving the antioxidant and anti-apoptotic effects of DATS in experimental models.

Experimental Model	Dose/Duration/Route	Molecular Mechanisms	Molecular Finding(s)	Molecular Effect(s)	Ref.
In vitro isolated H9c2 cardiomyocytes exposed to HG	33 mM	Activation of IGF1R/pAkt survival pathway	↑ CSE expression ↓ Bak and caspase 3 ↓ pIGF1R and p-AKT proteins ↓ NOX-2, p-47	↓ OS ↓ cellular apoptosis	[87]
In vitro isolated H9c2 cardiomyoblasts exposed to HG	10 µM	Activation of PI3K/Akt/Nrf signaling pathway	↑ Nrf2 protein expression ↑ Antioxidant enzymes (HO-1, SOD-1, SOD-2) ↓ Keap1, GSK3β expression ↓ apoptotic bodies, ↓cleaved-caspase 3		[88]
In vitro isolated H9c2 cardiomyoblasts and neonatal cardiomyocytes exposed to HG	1–10 µM	Inhibition of ROS-stimulated JNK/NF-κB signaling pathway	↓ JNK phosphorylation ↓ c-Jun phosphorylation ↓ apoptotic bodies ↓ caspase-3		[59]
In vitro H9c2 cardiomyoblasts exposed to HG I/R injury in DM rats	10 μM 6 h 20 mg/kg 3 days before IRI	Activation of AMPK-mediated AKT/GSK-3β/HIF-1α signaling pathway	↑ Bcl-2 expression ↓ cleaved caspase-3 and Bax expression	↓ cellular apoptosis	[62]
In vitro DOX-induced H9c2 cardiomyocytes In vivo DOX-induced rats	1, 5, 10 µM	Inhibition of ROS-dependent JNK/ERK/NFkB signaling pathway	↓ p22phox and p47phox protein levels ↓ phosphorylated JNK/ERK ↓ Bax, caspase 3		[25]
In vivo obese DM rats In vitro HUVECs exposed to HG	5.0 mg/kg/day i.v. 7 days 25–100 µmol/L 24 h	Preserved activity of mitochondrial antioxidant defense system (SOD, GSH-Px) * Suggested further research	↓ endothelial injury Improved endothelial maximal relaxation percent ↑ cell viability, ↓ LDH activity ↓ ROS, MDA in mitochondria ↑ NO bioavailability ↓ mitochondrial disfunction (↑ ΔΨm, ATP levels, and O_2_ consumption)	↓ mitochondrial OS and HG-induced endothelial injury	[89]
I/R injury in STZ-induced DM rats	40 mg/kg/day 3 days	Activation of SIRT-1 signaling pathway	↓ apoptotic index, ↓ caspase 3, ↓ cleaved caspase-3 ↓ p-PERK/PERK and p-eIF2α/eIF2α ratio ↓ ATF4, CHOP and caspase-12 Activation of Nrf-2/HO-1 signaling ↓ Nox-2 and Nox-4 protein expressions ↓ O_2_^−^, ↓ MDA, ↓ SOD	↓ OS and ER stress-induced cardiac apoptosis	[73]
Ex vivo I/R injury in STZ-induced DM rats	Per os by gavage 40 mg/kg body weight, every other day for 21 days	Inhibition of ROS and apoptosis	↓ O_2_^−^, ↑ NO_2_^−^ ↑ CAT, ↑ SOD ↓ tunel staining ↑ SOD-2 and Bcl-2 expression ↓ Bax and caspase-3 expression	↓ OS ↓ apoptosis	[63]
Ex vivo I/R injury in rats with metabolic syndrome	Per os by gavage 40 mg/kg body weight, every other day for 21 days	Inhibition of ROS, apoptosis, and inflammation	↓ TBARS, ↓ O_2_^−^, ↑ NO_2_^−^ ↑ CAT, ↑ GSH, ↑ SOD ↓ tunel staining ↓ picrosirius red and vimentin staining ↑ Bcl-2 and caspase-3 expression ↑ HSP70 ↓ Bax and caspase-9 expression ↑ SOD-1, ↑ SOD-2 ↓ NF-kB, TNF-alpha and IL-17A expression	↓ OS ↓ apoptosis ↓ inflammation	[64]
In vitro HUVECs exposed to LDL	50 µM	Activation of PI3/PKB-dependent eNOS signaling pathway	preserved caveolin-1-associated eNOS ↑ cGMP content ↓ chymotrypsin-like proteasome activity ↑ PKB phosphorylation	Improved eNOS activity and NO production	[90]

Abbreviations: HG—high glucose; I/R—ischemia/reperfusion; eNOS—endothelial nitric oxide synthase; DOX—doxorubicin; O_2_^-^—superoxide anion radical; MDA—malondialdehyde; TBARS—thiobarbituric acid reactive substances; NO_2_^−^—nitrites; SOD—superoxide dismutase; GSH—reduced glutathione; CAT—catalase; NF-kB—nuclear factor kappa-light-chain-enhancer of activated B cells; ↑—increased; ↓—decreased.
